# Effects of Different Dual-Modified Jujube Juicing Residue Dietary Fibers on the Properties of Egg Protein Gels Induced by Alkalinity and Heat

**DOI:** 10.3390/gels11060399

**Published:** 2025-05-27

**Authors:** Xinyu Zheng, Ling Dang, Yichan Zhang, Xinyu Liu, Hui Wang, Yajun Zheng, Xinling Song, Zhihui Wei, Jiayao Zhang, Xiaoyang Guo

**Affiliations:** 1 Food Science College, Shanxi Normal University, Taiyuan 030031, China; 18315884170@163.com (X.Z.); 13453217843@163.com (Y.Z.); 13935134643@163.com (X.L.); 18635530501@163.com (H.W.); 13546675098@163.com (X.S.); 17836564558@163.com (Z.W.); z15330839916@163.com (J.Z.); 13607692378@163.com (X.G.); 2 College of Health Management, Shanxi Technology and Business University, Taiyuan 030006, China; dangling1000@163.com

**Keywords:** jujube juicing residue dietary fibers, cellulase and xylanase hydrolysis, carboxymethylation, phosphate crosslinking, hydration properties, heat-induced and alkaline-induced egg protein gels, textural properties

## Abstract

Egg protein gels have relatively poor water-holding capacity, hardness, and freeze–thaw properties. Jujube juicing residue dietary fiber (JJRDF) is available, but it is rarely used in the food industry because of its poor hydration properties. Versions of JJRDF modified via cellulase and xylanase hydrolysis separately coupled with carboxymethylation (JJRDF-CXHC), phosphate crosslinking (JJRDF-CXHPC), and acetylation (JJRDF-CXHA) were prepared, and their effects on heat-induced and alkaline-induced egg protein gels (HA-EPGs) were studied. Smaller particle sizes and higher solubility, viscosity, expansion volume, and ability to retain water were observed in JJRDF-CXHC, JJRDF-CXHPC, and JJRDF-CXHA compared to JJRDF (*p* < 0.05). JJRDF-CXHC showed the highest viscosity (18.46 cP) and expansion volume (10.40 mL/g). Higher random coil and β-sheet contents resulted in an increase in pH, adhesiveness, hardness, and chewiness, and a decrease in the water-losing rate in freeze–thaw cycles, and gastric digestion was observed in the HA-EPGs as a consequence of adding JJRDF, JJRDF-CXHC, JJRDF-CXHPC, and JJRDF-CXHA at 3–5 g/100 g. Moreover, JJRDF-CXHC and JJRDF-CXHPC were better at improving the textural quality of the unmodified HA-EPG compared to JJRDF-CXHA and JJRDF (*p* < 0.05). Therefore, to improve egg protein gel quality, JJRDF modified with cellulase and xylanase hydrolysis separately coupled with carboxymethylation and crosslinking is a good choice. However, the functionalities of these modified JJRDFs should be studied.

## 1. Introduction

Red jujube (*Ziziphus jujuba* Mill.) is widely planted in north and northwest China, especially in Shanxi, Hebei, Henan, and Xinjiang. In addition to fresh and dry food, jujube is processed into jujube wine, juice, cake, and other products. During the processing of these products, substantial jujube juicing residues are generated, amounting to an annual yield of 30,000 tons. Jujube juicing residue contains high contents of dietary fiber (15 g/100 g), but due to its high content of insoluble cellulose and hemicellulose (84.9 g/100 g), most jujube juicing residue is discarded in China [1]. Prior studies have shown that the water-retention capacity of jujube juicing residue dietary fiber (JJRDF) is good, but its adsorption ability and hydration properties are low [2,3,4]. The predominant reason for this phenomenon is its low soluble fiber content (5 g/100 g) [5]. Furthermore, accumulating studies have revealed that soluble dietary fiber is better at preventing obesity, hyperglycemia, gastrointestinal inflammation, and cancer [6,7,8]. Soluble dietary fiber also plays an important role in the gel, emulsion, foaming, and antioxidant properties of fibers [9]. However, dietary fiber (DF) from most cereals and vegetables has low contents of soluble dietary fiber [10]. Therefore, it is necessary to increase the soluble fiber content of JJRDF and improve its hydration properties. Recently, some biological and chemical methods, such as enzymolysis, carboxymethylation, phosphate crosslinking, and hydroxylation, have been used to improve the polarity and functional properties of DF [8,9,10,11,12]; however, to the best of our knowledge, there are limited data on the synergistic effects of these modifications.

Proteins can form gel network structures after denaturation, the unwinding of the helix structure, and polypeptide chains’ re-aggregation [13,14]. Due to the biocompatibility, degradability, load capacity, and good emulsification, plasticity, and textural properties of protein gels, they have broad applications in the food, cosmetics, and medical industries, especially egg white protein gels (EWPG) [15,16]. Egg white proteins contain ovalbumin (54.2 g/100 g), ovotransferrin (12 g/100 g), ovomucin (14.5 g/100 g), and lysozyme (3.4 g/100 g) [17]. Under heating, egg protein denaturants and their α-helix, β-turn, and random coil are depolymerized, and functional groups (such as sulfhydryl) are exposed; following this stage, under alkaline action, the polypeptide chains reassemble through disulfide bonds, hydrophobic forces, and electrostatic repulsion, and they form gel network structures [18]. In recent years, modified egg white gels have exhibited potential applications in areas such as biotissue engineering, skin-care masks, bionic electronic skin, wearable devices, and stimulus-responsive actuators [19].

Egg protein gels formed using heat and alkalinity (HA-EPGs) have good plasticity and digestibility, and they have a special taste and flavor; however, the fragility and relatively poor hardness, chewiness, springiness, freeze–thaw property, and dark color limit their application [16,20]. The addition of polysaccharides has been proven to enhance the hardness, chewiness, and springiness of egg protein gels [21,22]. Corn starch improves the hardness and springiness of egg protein gels by forming hydrogen bonds with egg proteins. Moreover, the disulfide bonds, hydrophobic forces, and ionic bonds between proteins are enhanced by the addition of polysaccharides [23,24].

Dietary fiber is a type of polysaccharide that cannot be digested and absorbed in the stomach and small intestine, and it plays a crucial role in human health [25]. Dietary fiber, especially soluble fiber, can effectively improve the interactions between egg white proteins and the hydrogen bonds, disulfide bonds, and water transport forces within and between the polypeptide chains, thereby improving the gel and emulsion properties and the textural quality of egg white proteins [26,27]. Apart from this, the addition of dietary fiber endows the proteins with functional properties, such as hypoglycemic properties, improving the gastrointestinal flora balance, anticancer and hypolipidemic properties, and heavy metal adsorption of egg white proteins [28,29]. By contrast, due to steric hindrance, dilution effects, and physical steric effects, the addition of insoluble dietary fibers can weaken the hydrogen, disulfide, and ionic bonds within egg polypeptide chains, thereby decreasing the amount of protein gels, such as is the case with tofu and gelatin [9].

Recently, due to its poor hydration properties and harsh flavor, JJRDF has not been used in protein hydrogels. Cellulase and xylanase hydrolysis have been successfully applied to improve the functionality of dietary fibers [11,30]. The physicochemical and functional properties of coconuts, millet bran, and oat dietary fibers are effectively improved via carboxymethylation, acetylation, or crosslinking [7,31]. However, there are limited data regarding the synergistic effects of cellulase and xylanase hydrolysis combined with carboxymethylation, acetylation, or crosslinking with respect to JJRDF. If cellulase and xylanase hydrolysis combined with carboxymethylation, acetylation, or crosslinking can improve the hydration and property of JJRDF, addition of the modified JJRDF will improve the textural properties of HA-EPGs and expand their applications. Therefore, one purpose of this study is to investigate the effects of cellulase and xylanase hydrolysis separately united with carboxymethylation, phosphate crosslinking, and acetylation on the solubility and hydration properties of JJRDF. Another purpose is to study the influence of addition of the modified JJRDFs on the textural properties of HA-EPGs. This study aims to provide new strategies for enhancing egg white protein gels formed via heat and alkaline treatment.

## 2. Results and Discussion

### 2.1. Effect of Dual Modifications on Chemical Composition of JJRDFs

One purpose of biological or chemical modification is to increase the purity and SDF contents of plant dietary fibers; this is because an increase in SDF can enhance the interactions between dietary fibers and water or oil molecules and thus improve their hydration or lipid-referring properties and functionalities [32]. As shown in Table 1, after cellulase and xylanase hydrolysis with further unification with carboxymethylation, phosphate crosslinking, or acetylation, the substitution degrees of JJRDF-CXHC, JJRDF-CXHA, and JJRDF-CXHPC were 3.52%, 3.84%, and 1.19%, respectively, showing that the carboxymethyl, acetyl, and phosphate groups were grafted in the JJRDF. As a consequence, the SDF content of JJRDF-CXHC, JJRDF-CXHA, and JJRDF-CXHPC was higher than that of JJRDF, and their insoluble fiber content was correspondingly decreased (*p* < 0.05). Cellulase and xylanase degraded the glycosidic bonds in dietary fiber and released polar chemical groups [26]; in contrast, the grafting of carboxymethyl and phosphate groups has been proven to effectively improve dietary fiber polarity [7], resulting in higher JJRDF hydrophilicity and solubility. Furthermore, JJRDF-CXHC, JJRDF-CXHA, and JJRDF-CXHPC exhibited higher SDF contents than JJRDF (*p* < 0.05), indicating that dual enzymolysis alone was less effective for increasing polarity than dual enzymolysis combined with chemical modifications. Similar results were obtained by Mohammadi et al. [30]. Additionally, a lower SDF content was observed in JJRDF-CXHA compared with that of JJRDF-CXHC and JJRDF-CXHPC (*p* < 0.05), which was ascribed to the stronger polarity of the carboxymethyl and phosphate groups than of the acetylic group [31]. Alternatively, these dual modifications did not have an obvious impact on the protein, moisture, and fat contents of JJRDF (*p* > 0.05), but the ash content was increased, mainly attributed to the application of chemical reagents during chemical modifications.

### 2.2. Colour and Size of JJRDFs

The color difference (*∆E*) between the samples is visible to the naked eye if the *∆E* value reaches 4 [33]. The results in Table 1 show that JJRDF-CXHA, JJRDF-CXHC, and JJRDF-CXHPC exhibited visible color differences (with higher *a* or *b* values and lower *L* value) compared to JJRDF (*p* < 0.05). Of these, JJRDF-CXHC exhibited the highest *∆E* and lowest *L* value. As *L* and *b* values represented lightness and yellowness, these findings revealed that the color of JJRDF darkened after the dual modifications. During dual enzymolysis with chemical modifications, heating, basic treatment, carboxymethylation, acetylation, and/or crosslinking resulted in the oxidative browning reaction of JJRDF, further resulting in a decrease in its lightness [34]. A larger particle size indicates a smaller interacting area of DF with water and oil and may deteriorate gels’ textural quality [25]. The D_3,2_ values of JJRDF-CXHA, JJRDF-CXHC, and JJRDF-CXHPC were lower than that of JJRDF, and their surface area was larger, which was a result of the degradation of glycosidic bonds caused by cellulase and xylanase hydrolysis and carboxymethylation, acetylation, or crosslinking [26,30]. JJRDF-CXHC had a larger surface area than JJRDF-CXHA and JJRDF-CXHPC, confirming that carboxymethylation was better at reducing the particle size of JJRDF than acetylation and crosslinking. Similar results were obtained by Mohammadi et al. [30] and Zheng and Li [25].

### 2.3. Structural Characteristics

#### 2.3.1. Scanning Electron Microscopy of JJRDFs

**Figure 1:**
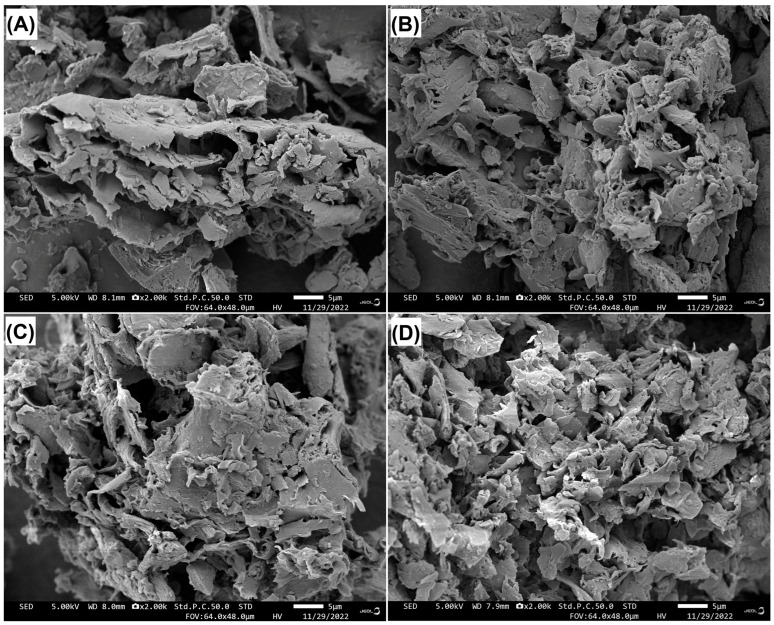
Scanning electron micrographs of JJRDF (**A**), JJRDF-CXHC (**B**), JJRDF-CXHPC (**C**), and JJRDF-CXHA (**D**), with a magnification of 2000×, at 5 μm. JJRDF, jujube juicing residue dietary fiber; JJRDF-CXHC, JJRDF modified via cellulase and xylanase hydrolysis united with carboxymethylation; JJRDF-CXHPC, JJRDF modified via dual enzymolysis united with phosphate crosslinking; JJRDF-CXHA, JJRDF modified via dual enzymolysis united with acetylation.

The electron microscopy scanning pictures in Figure 1A–D reveal that JJRDFs had porous and fragmented microscopic surface structures, resembling the classic microstructure of DF rich in lignin and hemicellulose [35]. There are more pores and fragments in the scanning pictures of JJRDF-CXHA, JJRDF-CXHC, and JJRDF-CXHPC (Figure 1B–D) in comparison to that of JJRDF (Figure 1A). This is ascribed to the degradation of glycosidic bonds caused by cellulase and xylanase hydrolysis and reductions in hemicellulose and cellulose (Table 1) [31]. During carboxymethylation, alkalization and heating resulted in the degradation of DF chains, leaving more holes or cracks [11]. The increase in the number of holes and debris was consistent with the smaller particle size of JJRDF-CXHA, JJRDF-CXHC, and JJRDF-CXHPC (Table 1), and this was in line with their hydration properties and adsorption capacities [6].

#### 2.3.2. Fourier-Transform Infrared Spectra

**Figure 2:**
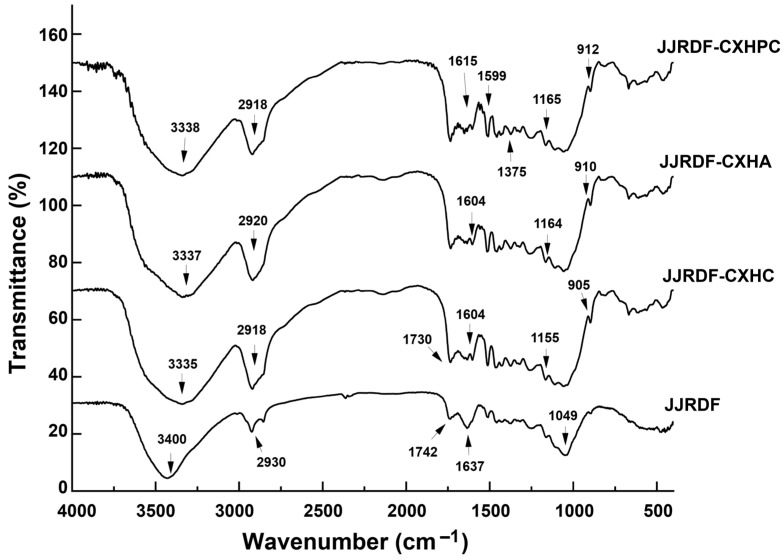
Fourier-transformed infrared spectroscopy of JJRDF, JJRDF-CXHC, JJRDF-CXHPC, and JJRDF-CXHA.

As shown in Figure 2, slight differences were observed between the FI-IR spectra of JJRDF, JJRDF-CXHA, JJRDF-CXHC, and JJRDF-CXHPC. After xylanase and cellulase hydrolysis was carried out, coupled with carboxymethylation, acetylation, or crosslinking, the branded peak at 3400 cm^−1^ in the spectrum of JJRDF shifted to 3335, 3337, and 3338 cm^−1^, and this phenomenon is ascribed to the asymmetric stretching of hydrogen bonds [17]. The new peaks that appeared at 905, 910, and 912 cm^−1^ in the spectra of JJRDF-CXHC, JJRDF-CXHA, and JJRDF-CXHPC, were a consequence of the vibration of β-C-H and revealed that β-glycosidic bonds in JJRDF were cracked during these dual modifications [30]. The blue shift (from 1149 to 1155 cm^−1^) and red-shift (from 1742 to 1730 cm^−1^) revealed the deformation of the methylene and carboxyl groups in JJRDF, respectively, verifying the grafting of carboxymethyl [33]. Furthermore, the phosphate groups were linked to JJRDF because a new peak appeared at 1375 cm^−1^ within the spectrum of JJRDF-CXHPC. The vibration of the P=O bond resulted in an adsorption peak at approximately 1370 cm^−1^ in the FI-IR spectra of the DF [21]. Additionally, the grating of acetyl groups in JJRDF can be revealed via the red shift (from 2930 to 2910 cm^−1^) in the spectrum of JJRDF-CXHA [6]. These findings demonstrate that the structure of the JJRDF, especially its chemical groups and linking bonds, was altered by those dual modifications.

### 2.4. Viscosity, ARW, and EVW of JJRDFs

Viscosity and expansion volume in water are crucial indices for evaluating the capacity of DF in improving food textural properties; furthermore, a high capacity to retain water means that DF can effectively enhance the quality and shelf-life of hydrogels [8]. As shown in Table 1, the viscosity, ARW, and EVW of JJRDF-CXHC, JJRDF-CXHPC, and JJRDF-CXHA were higher than those of JJRDF (*p* < 0.05); this is predominately ascribed to the higher SDF content, the larger surface area (Table 1), and the grafting of acetyl, carboxymethyl, and phosphate groups. Soluble fiber can improve the affinity of DF to water, resulting in higher hydrophilicity and viscosity [21]. A decrease in particle size will expand the effective contact area between DF and waters, and the holes and debris (Figure 1B–D) are helpful for DF to capture and retain water [6]. Additionally, the hydrophilicity of carboxymethyl and the induced phosphate groups may increase the steric hindrance between fiber chains and expand the volume of DF in water [35].

Moreover, the reasons for the high ARW and WSA of JJRDF-CXHPC included the network structure between the fibers’ chains formed after phosphate crosslinking, which retained more water molecules [33]. The high WSA of JJRDF-CXHA was consistent with it having the largest surface area (Table 1) [26]. In addition, the introduction of the acetyl group can enhance steric hindrance across polysaccharide chains, thus rendering the structure of cellulose molecules more relaxed in aqueous solutions [31]. JJRDF-CXHC exhibited the highest viscosity (18.46 cP), and this phenomenon is due to it having the greatest expansion volume in water and the highest water retention ability (Table 1), and the hydrophilicity of carboxymethyl groups [33]. In contrast with JJRDF-CXHA, a higher viscosity and ability to retain water, and a greater water expansion volume, were observed in JJRDF-CXHC and JJRDF-CXHPC (*p* < 0.05); this is attributed to the lower hydrophilicity of the acetyl group and the relatively poor SDF content of JJRDF-CXHA. Zhang and Ye [1] modified jujube kernel fiber via crosslinking and the modified product exhibited lower viscosity and expansion volume in water, confirming that xylanase and cellulase hydrolysis combined with crosslinking were better at enhancing the hydration properties of DF.

### 2.5. HA-EPG Structure

#### 2.5.1. Scanning Electron Microscopy

**Figure 3:**
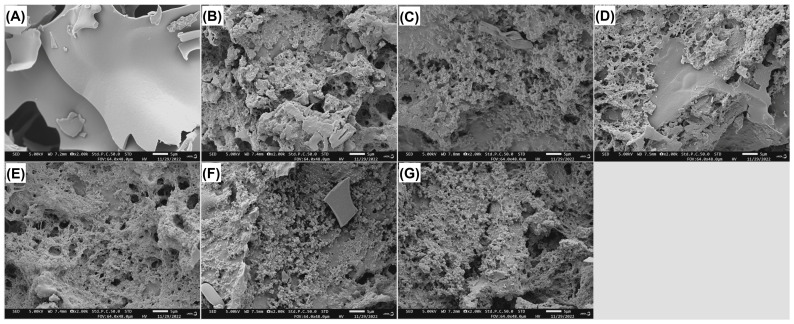
Scanning electron micrographs of heat- and alkaline-induced egg protein gels (**A**); heat- and alkaline-induced egg protein gels fortified with JJRDF at 5 g/100 g (**B**); heat- and alkaline-induced egg protein gels fortified with JJRDF-CXHA at 5 g/100 g (**C**); heat- and alkaline-induced egg protein gels fortified with JJRDF-CXHPC at 5 g/100 g (**D**); heat- and alkaline-induced egg protein gels fortified with JJRDF-CXHC at 1 g/100 g (**E**); heat- and alkaline-induced egg protein gels fortified with JJRDF-CXHC at 3 g/100 g (**F**); and heat- and alkaline-induced egg protein gels fortified with JJRDF-CXHC at 5 g/100 g (**G**) with a magnification of 2000×, at 5 μm.

Figure 3B–D,G show the electron microscopy scanning pictures of heat- and alkaline-induced egg protein gels containing 5 g/100 g of JJRDF, JJRDF-CXHA, JJRDF-CXHC, and JJRDF-CXHPC, respectively. Compared to the smooth microstructural surface of the unmodified HA-EPG (Figure 3A), there were many holes and debris in the structure of HA-EPG/JJRDF, HA-EPG/JJRDF-CXHA, HA-EPG/JJRDF-EPC, and HA-EPG/JJRDF-CXHC (Figure 3B–D,G). The addition of fibers has a physical steric effect on the aggregation of egg polypeptide chains [34]; in addition, fibers can be used as a skeleton during gel formation, which is helpful for the aggregation of egg polypeptide chains [14,28,36], resulting in granular and porous microstructures. Liu et al. [24] and Li et al. [22] obtained similar results. The results in Figure 3E,F reveal that as the dose of JJRDF-CXHC increased from 1 to 3 g/100 g, the holes and granules became smaller, and the microstructural surfaces of the HA-EPGs became smoother and denser, demonstrating that the intramolecular and intermolecular aggregation of egg polypeptide chains were enhanced via the addition of JJRDF-CXHC. The high viscosity and ability to retain water were helpful for JJRDF-CXHC relative to improving the affinity of egg proteins for water; moreover, the carboxymethyl groups enhanced the intramolecular attraction of the egg polypeptide chains [37]. However, the holes and granules in the microstructural surfaces of the HA-EPGs increased when the dose of JJRDF-CXHC increased to 5 g/100 g (Figure 3G); this is likely a result of the physical steric effect of JJRDF-CXHC relative to egg polypeptide chains [38].

#### 2.5.2. Secondary Structure

**Figure 4:**
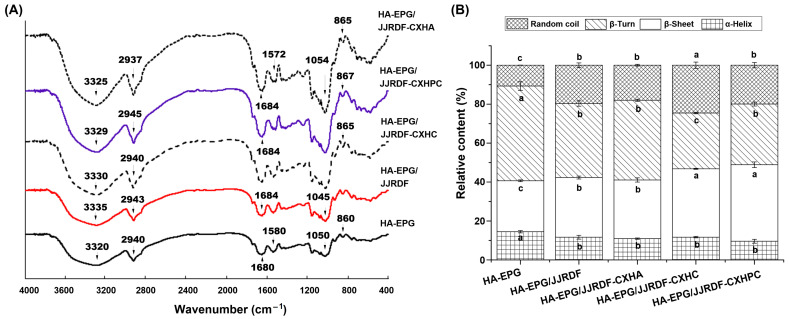
Fourier-transform infrared spectroscopy (**A**) and relative content of the protein secondary structure (**B**) of HA-EPG, HA-EPG/JJRDF, HA-EPG/DEC, HA-EPG/JJRDF-HDEPC, and HA-EPG/JJRDF-CXHA. HA-EPG, heat- and alkaline-induced egg white protein gel; HA-EPG/JJRDF, heat- and alkaline-induced egg white protein gel with JJRDF; HA-EPG/JJRDF-CXHC, heat- and alkaline-induced egg white protein gel with JJRDF-HDEC; H-EWP/JJRDF-CXHPC, heat- and alkaline-induced egg white protein gel with JJRDF-CXHPC; and H-EWP/JJRDF-CXHA, heat- and alkaline-induced egg white gel with JJRDF-CXHA.

All HA-EPGs exhibited similar FT-IR spectra, but an obvious shift was observed in several characteristic peaks (Figure 4A). Of these, the red shift in peak at 3320 cm^−1^ exhibited changes in the amide band I of HA-EPG, which are a result of the stretching vibration of the N-H or O-H group after the addition of JJRDFs [39]. The peak at 1680 cm^−1^ transferred to 1684, 1684, 1691 and 1687 cm^−1^ after the HA-EPGs were fortified with JJRDF, JJRDF-CXHC, JJRDF-CXHPC, and JJRDF-CXHA, respectively, verifying that those changes occurred in the amide band II of HA-EPG [22]. Moreover, transfers occurred in the peaks at 1050 and 860 cm^−1^, reflecting the stretching vibration of ether bonds and the symmetric vibration of glycosidic bonds [38], respectively; this demonstrates the introduction of polysaccharide bonds into egg white proteins after the addition of JJRDFs.

Based on the results of Figure 4A, PeakFit software v4.12 was used to analyze the specific changes in the secondary structures of the HA-EPGs. As shown in Figure 4B, improvements in the effect on the β-sheet and random coil contents of HA-EPG and a reduction in influence relative to theβ-turn and α-helix contents were found in JJRDF, JJRDF-CXHC, JJRDF-CXHPC, and JJRDF-CXHA (*p* < 0.05), corresponding to the changes in the amide band I and II of HA-EPG, respectively (Figure 4A) [37]. The physical steric effect of these JJRDFs can enhance the space barrier between egg polypeptide chains. Moreover, the addition of JJRDFs can improve the repulsive forces between egg polypeptide chains, which is conducive to the formation of random coil in HA-EPGs [16]. On the other hand, JJRDFs with considerable hydration properties (ability to restrain water and viscosity, Table 1) can improve the hydrophilicity of egg proteins and thereby promote the formation of hydrogen bonds between egg polypeptide chains [32]. An increase in hydrogen bonds means that a higher β-sheet content will be present in egg protein gels [39]. Furthermore, JJRDF-CXHPC and JJRDF-CXHC exhibited a better increasing effect relative to the β-sheet content of HA-EPG than JJREDF-CXHA, which is primarily due to the high hydrophilicity of the phosphate and carboxymethyl groups and the higher hydration properties of JJRDF-CXHPC and JJRDF-CXHC (Table 1). Hou et al. [33] found that soybean fibers increased the β-sheet content in konjac glucomannan/κ-carrageenan gel.

### 2.6. Effect of the Addition of JJRDFs on the Morphological Diagram and Color of HA-EPGs

**Figure 5:**
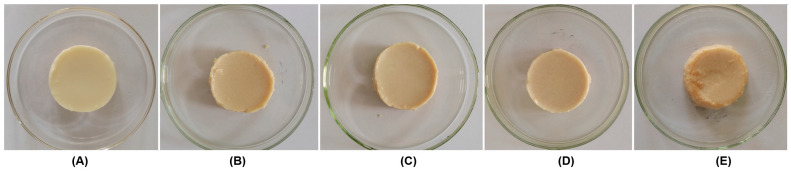
Morphological diagram of the heat- and alkaline-induced egg protein gel (**A**), and heat- and alkaline-induced egg protein gel containing 3 g/100 g of JJRDF (**B**), JJRDF-CXHA (**C**), JJRDF-CXHC (**D**), and JJRDF-CXHPC (**E**).

The morphological diagrams of the heat- and alkaline-induced egg protein gel containing 5 g/100 g of JJRDF, JJRDF-CXHA, JJRDF-CXHC, and JJRDF-CXHPC are shown in Figure 5A–E. It is obvious that the addition of JJRDFs changed the color and morphological diagram of the unmodified HA-EPG, which showed a smooth surface (Figure 5A); the surface then became rough after the addition of JJRDFs (Figure 5B–E), corresponding to their scanning electron micrographs (Figure 3). Moreover, the color of the HA-EPGs with JJRDF (B), JJRDF-CXHA (C), JJRDF-CXHC (D), and JJRDF-CXHPC (E) became darker with higher *a* and *b* values and lower *L* value (Table 2). One reason for this phenomenon is that these JJRDFs (containing a high content of insoluble dietary fiber, Table 1) have a blocking effect on the light transmission of the HA-EPGs, darkening their color [12]; another reason is that the darker color of JJRDFs reduced the lightness of the HA-EPGs, also resulting in darkening. Similar results were obtained by Ullah et al. [21].

### 2.7. Physicochemical Properties of HA-EPGs

#### 2.7.1. Ability of HA-EPGs to Retain Water

The ARW of hydrogels affects their structural properties and applications in foods, especially with respect to food preservation and moisturizing [12]. At a dose of 3 g/100 g, obvious improving effects on the ARW of the unmodified HA-EPG were observed in JJRDF, JJRDF-CXHC, and JJRDF-CXHPC. JJRDF-CXHPC, JJRDF-CXHC, and JJRDF have a physical steric effect on egg protein aggregation during heat- and alkaline-induced hydrogels [31], which can increase the gap between the gels (Figure 1) and thus enhance the ARW of HA-EPG. As JJRDF, JJRDF-CXHC, and JJRDF-CXHPC exhibited relatively high hydration properties (Table 1), they can increase the hydrophilicity of egg proteins, thereby improving the ARW of HA-EPG. HA-EPG/JJRDF-CXHC showed the highest ARW at 3g/100 g; this is because the carboxymethyl groups in JJRDF-CXHC can significantly improve the binding force between egg proteins and water [20]. As the dose increased to 5 g/100 g, the ARW of HA-EPG/JJRDF, HA-EPG/JJRDF-CXHC, and HA-EPG/JJRDF-CXHPC decreased remarkably, probably because the addition of a large number of JJRDFs (mainly comprising insoluble fiber) reduced the hydrophilicity of the egg protein gels and decreased the affinity of the egg protein chains for water [28]. These findings can explain why HA-EPG/JJRDF-CXHC showed a denser microstructure at 3 g/100 g than at 5 g/100 g (Figure 3E–G). In contrast, JJRDF-CXHA had a reducing effect on the ARW of HA-EPG at 1–5 g/100 g, predominantly due to the acetyl groups in JJRDF-CXHA. A prior study found that the acetylation of proteins caused the aggregation of egg polypeptide chains and enhanced the hydrophobic force between proteins [20], resulting in a reduction in the ability of HA-EPGs to hold water. An increment in ARW means that the modified HA-EPGs can be used in meat products and bread to improve their water-holding capacity and textural quality [14].

#### 2.7.2. pH Values

**Figure 6:**
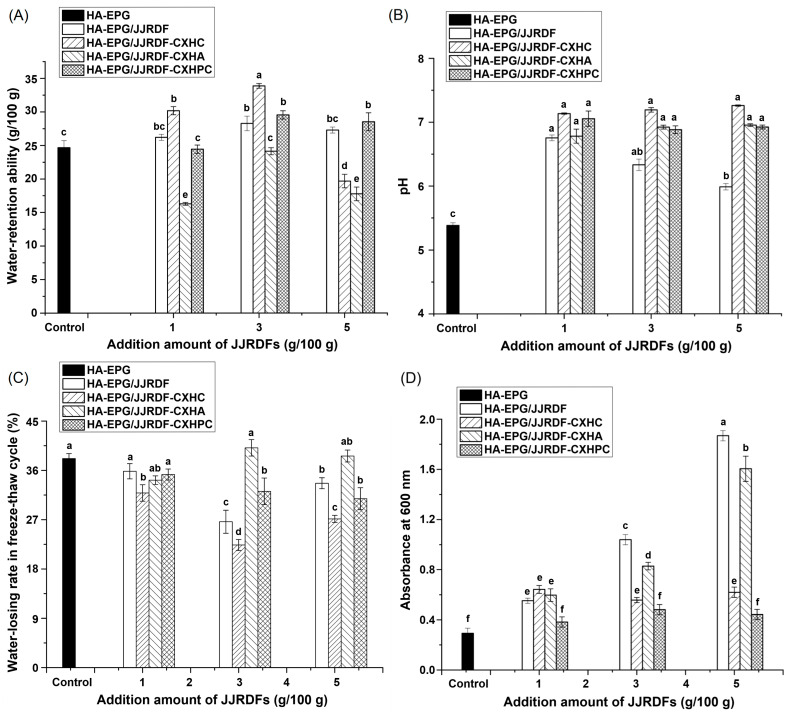
Effects of different dose of JJRDFs on the water-retention ability (**A**), pH (**B**), water-losing rate in freeze–thaw cycle (**C**), and optical transparency (**D**) of heat-induced egg white protein gel (H-EPG). Different lower letters (a–f) on the data points denote significant differences (*p* < 0.05).

At the isoelectric point (pH 5.4 ± 0.2), the electrostatic repulsion between egg proteins is zero and the egg proteins will clump together and then precipitate out of the solution, forming a gel during cooling; however, the HA-EPG that formed at the isoelectric point showed relatival poor textural and sensory qualities because of the excessive aggregation of polypeptide chains [14]. JJRDF, JJRDF-CXHC, JJRDF-CXHPC, and JJRDF-CXHA (1–5 g/100 g) exhibited remarkably enhancing impacts on the HA-EPGs’ pH values (*p* < 0.05) because these JJRDFs inflict dilution effects on egg polypeptides, enhancing the ARW of HA-EPGs (Figure 6A). During the formation of heat- and alkaline-induced hydrogels, the addition of fibers can increase the affinity of egg polypeptide chains for water and cause a dissociation of amino groups in egg proteins [13], resulting in increments in the pH values of the HA-EPGs. Moreover, an increase in pH value (6.0–8.5) is helpful for the formation of a uniform network gel structure, which is an explanation for HA-EPGs’ higher ability to retain water (Figure 6A). Zhao et al. [23] found that the addition of soybean dietary fibers improved the pH of egg protein gel as well.

#### 2.7.3. Water-Losing Rate After Freeze–Thaw

A high water-losing rate during freeze–thaw cycles means that hydrogels can sustain their water content and structural quality, avoiding shrinkage and efficacy loss [14]. As shown in Figure 6C, the water-losing rate of HA-EPG was notably reduced via the addition of JJRDF, JJRDF-CXHC, and JJRDF-CXHPC at 3–5 g/100 g (*p* < 0.05). As dietary fibers have physical steric effects on egg polypeptide chains during hydrogels formation, increasing the number of holes in the microstructure (Figure 3B–E), which is conducive for ice crystal storage and water-retention during freeze–thaw cycles [33]. Moreover, the addition of JJRDF, JJRDF-CXHC, and JJRDF-CXHPC increased the random coil and β-sheet contents of HA-EPG (Figure 4B) and enhanced their ability to retain water (Figure 6A), resulting in a lower water-losing rate during freeze–thaw cycles. Similar findings were obtained by Xu et al. [31] and Tian et al. [34]. Furthermore, the lowest water-losing rate was observed in HA-EPG/JJRDF-CXHC, corresponding to HA-EPG/JJRDF-CXHC’s ability to retain water (which is the highest of the samples), its β-sheet and random coil contents (Figure 4B and Figure 6A), and its excellent hydration properties (Table 1). Carboxymethyl groups can increase the hydrophilicity of egg polypeptide chains and improve the structure of HA-EPG, leading to a good water-holding abilities during freeze–thaw cycles. Additionally, the water-losing rates of HA-EPG/JJRDF and HA-EPG/JJRDF-CXHC at 5 g/100 g were lower than those at 3 g/100 g. Relative to the addition amount of 5 g/100 g, although JJRDF and JJRDF-CXHC increased the hydrophilicity of HA-EPG, the physical steric effect of JJRDF and JJRDF-CXHC reduced the aggregation of egg polypeptide chains [24], resulting in a microstructure with many tiny holes and cracks (Figure 3F), which is not conducive to water-retention during freeze–thaw cycles. A decrease in water-losing rates during freeze–thaw cycles indicates that the modified HA-EPGs can be used in frozen foods or skincare masks to sustain their water content and structural quality, avoiding shrinkage and efficacy loss [33].

#### 2.7.4. Light Transmittance

The light transmittance of gels is inversely proportional to their absorption at 600 nm [38]. As shown in Figure 6D, compared to the unmodified HA-EPG, the higher absorbance of HA-EPGs with JJRDF, JJRDF-CXHC, and JJRDF-CXHA (1–5 g/100 g) at 600 nm confirmed that JJRDF, JJRDF-CXHC, and JJRDF-CXHA lowered the optical transparency. After the addition of JJRDF, JJRDF-CXHC, and JJRDF-CXHA, the pH value of the HA-EPGs was increased (Figure 6B), the attraction force between egg proteins and waters was improved (Figure 6A), and the hydrophobic force between egg polypeptide chains were weakened, resulting in granular structures with tiny holes (Figure 3B–D,F). The structure of HA-EPGs is not conducive to the transmission of light, resulting in an absorbance increment at 600 nm [32]. Moreover, insoluble dietary fiber particles have a blocking effect on the light transmission of HA-EPGs, which is one reason for the dose-dependent reducing effect of JJRDF, JJRDF-CXHC, and JJRDF-CXHA on the light transmittance of HA-EPGs. The reduction in light transmission rendered HA-EPGs darker, but it is conducive for food preservation when HA-EPGs are used as a cling wrap [12]. In contrast, the addition of JJRDF-CXHPC did not alter the light transmittance of the HA-EPGs (*p* < 0.05), mainly because of the relatively denser microstructure of HA-EPG/JJRDF-CXHPC (Figure 3D). However, more research is needed to clarify the specific influence mechanism of these modified JJRDFs on the physicochemical properties of HA-EPGs.

### 2.8. Textural Qualities

As shown in Table 3, the hardness of the unmodified HA-EPGs increased with increasing addition doses of JJRDF, JJRDF-CXHA, JJRDF-CXHC, and JJRDF-CXHPC, and the highest hardness value was observed in HA-EPG/JJRDF-CXHPC at 5 g/100 g. After adding these modified JJRDFs, the hydrophilicity of the egg proteins was enhanced and the repulsive force between egg polypeptide chains was weakened [22]. Consequently, the aggregation of egg proteins was improved, resulting in an increment of hardness in the HA-EPGs. Moreover, these JJRDFs can serve as junction points or skeletons for egg polypeptide chains, which are helpful for the formation of a uniform and dense gel structure with high hardness values [15]. The higher water-retention ability of HA-EPG/JJRDF-CXHA, HA-EPG/JJRDF-CXHC, and HA-EPG/JJRDF-CXHPC (Figure 6A) contributed to their hardness as well. In addition, as the carboxymethyl, acetyl, and phosphate groups in JJRDF-CXHC, JJRDF-CXHA, and JJRDF-CXHPC, especially the phosphate groups, have been proven to remarkably enhance the crosslinking of biomacromolecules [29,31], HA-EPGs with these JJRDFs exhibited higher hardness.

The adhesiveness of the unmodified HA-EPG was increased via the addition of JJRDF, JJRDF-CXHC, and JJRDF-CXHA at 1–5 g/100 g (*p* < 0.05); in contrast, JJRDF-CXHPC did not alter the adhesiveness of HA-EPGs (*p* > 0.05). The addition of JJRDF, JJRDF-CXHC, and JJRDF-CXHA can enhance the interactions between egg polypeptide chains and increase the affinity of HA-EPGs for water, improving the aggregation of egg polypeptide chains [15] and increasing the adhesiveness of HA-EPGs. However, the addition of JJRDF-CXHPC may cause the excessive aggregation of egg polypeptide chains, and it remarkably increased the hardness of HA-EPGs, resulting in lower adhesiveness. Prior studies found that excessive hardness was not conducive to the adhesiveness of hydrogels [11,16].

Moreover, increases in the chewiness and gumminess of the unmodified HA-EPG were observed after the addition of JJRDF, JJRDF-CXHC, JJRDF-CXHPC, or JJRDF-CXHA (3–5 g/100 g) (*p* < 0.05). The gumminess of hydrogels was dependent on their viscosity, adhesiveness, and hardness; moreover, hardness, gumminess, and springiness played crucial role in the chewiness of hydrogels [15]. As the addition of JJRDF, JJRDF-CXHC, JJRDF-CXHPC, and JJRDF-CXHA improved the hardness and adhesiveness of the HA-EPGs (Table 3), consequently, gumminess increased correspondingly. Additionally, the considerable viscosity of JJRDF, JJRDF-CXHC, JJRDF-CXHPC, and JJRDF-CXHA (10.56–18.48 cP, Table 1) indicated that they can enhance the gumminess of HA-EPGs. Ascribed to the increased gumminess and hardness, HA-EPG/JJRDF, HA-EPG/JJRDF-CXHA, HA-EPG/JJRDF-CXHC, and HA-EPG/JJRDF-CXHPC exhibited higher chewiness than HA-EPG (*p* < 0.05). HA-EPG/JJRDF-CXHPC exhibited the highest chewiness and gumminess, followed by HA-EPG/JJRDF-CXHC; this is ascribed to their elevated hardness and ability to retain water (Table 3 and Figure 6A), and the viscosity, soluble fiber content, and higher random coil and β-sheet contents of JJRDF-CXHPC and JJRDF-CXHC (Table 1 and Figure 4B) [22,28]. Furthermore, the addition of JJRDF, JJRDF-CXHC, JJRDF-CXHPC, and JJRDF-CXHA did not notably alter the springiness and cohesiveness of the HA-EPGs (*p* > 0.05). In general, the improvement in hardness, chewiness, adhesiveness, and gumminess increases the sensory properties of HA-EPGs and expands their applications: for example, they can be used in thickeners, stabilizers, skincare masks, biotissue engineering, and bionic electronic skin [19].

### 2.9. Simulated Gastrointestinal Hydrolysis of HA-EPGs

**Figure 7:**
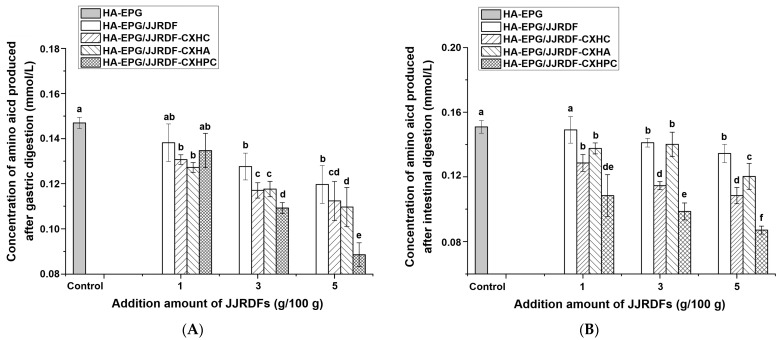
Effects of different dose of JJRDF, JJRDF-CXHC, JJRDF-CXHPC, and JJRDF-CXHA on the free amino acid production of heat-induced egg white protein gel under gastric (**A**) and intestinal (**B**) digestion. Different lowercase letters (a–f) on the data points indicate significant differences (*p* < 0.05).

Figure 7A,B depict the free amino acid content produced from the simulated gastric and intestinal hydrolysis of HA-EPGs, respectively. As shown in Figure 7A, a significant decrease in the production rate of the free amino acid of HA-EPG was found in JJRDF, JJRDF-CXHA, JJRDF-CXHC, and JJRDF-CXHPC (3–5 g/100 g), verifying that HA-EPG gastric hydrolysis was restrained by these JJRDFs. First, these JJRDFs have a physical steric effect on the interactions between HA-EPGs and gastric proteases [39]; second, these JJRDFs can increase pH value during gastric digestion (Figure 6B); third, these JJRDFs increased the hardness, gumminess, and chewiness of HA-EPGs (Table 3); fourth, JJRDF cannot be digested by gastrointestinal proteases, and these effects result in a slow production rate of free amino acid from the gastric hydrolysis of HA-EPGs (*p* < 0.05). With the highest hardness, chewiness, and gumminess values (Table 3), HA-EPG/JJRDF-CXHPC exhibited the lowest gastric hydrolysis rate. Additionally, the production rate of free amino acid from HA-EPG intestinal digestion was decreased via the addition of JJRDF, JJRDF-CXHPC, JJRDF-CXHC, and JJRDF-CXHA (at 3 and 5 g/100 g) (*p* < 0.05), which is predominately attributed to their enhancing effects on the hardness, gumminess, and chewiness of HA-EPGs (Table 3). Similar results were obtained by Xu et al. [31] and Lee et al. [38]. When the modified HA-EPGs are used as a carrier of active substances, a decrease in gastric digestibility means that the HA-EPG active substance conjugate exhibits relatively strong stability against gastric digestion, which is conducive to the stability of the active substance in vivo [16]. Therefore, a decrease in gastric hydrolysis is helpful for expanding the applications of HA-EPGs as bioactive substances carriers.

## 3. Conclusions

Modified jujube juicing residue dietary fibers (JJRDF-CXHA, JJRDF-CXHC, and JJRDF-CXHPC) were prepared via xylanase and cellulase digestion coupled with acetylation, carboxymethylation, and crosslinking, respectively. The obviously improved effects on JJRDF’s soluble fiber content (11.01–17.53 g/100 g), specific surface area (86.44–167.35 m^2^∙kg^−1^), ability to retain water (12.83–14.75 g/g), EVW (8.42–10.40 mL/g), and viscosity (10.56–18.46 cP) were observed in these dual modifications, and JJRDF-CXHA, JJRDF-CXHC, and JJRDF-CXHPC exhibited more porous microstructures. JJRDF-CXHC reflected the smallest particle size and highest viscosity (18.46 cP) and expansion volume in water (10.40 mL/g). More granular microstructures with tiny holes, higher random coil and β-sheet contents, an increase in pH, adhesiveness (from −47.32 to −26.21), hardness (from 93.16 to 201.41 g), and chewiness (from 80.14 to 177.99 g), and a decrease in water-losing rate (from 36.01% to 21.62%) during freeze–thaw cycles, light transmittance, and gastric digestion were observed in HA-EPGs modified with JJRDF, JJRDF-CXHC, JJRDF-CXHPC, and JJRDF-CXHA at 3–5 g/100 g. Moreover, JJRDF-CXHC and JJRDF-CXHPC were better at improving the water-retention ability (32.83–34.62 g/g) and textural quality of HA-EPGs than JJRDF-CXHA and JJRDF (*p* < 0.05).

These results suggest that xylanase and cellulase digestion coupled with acetylation, carboxymethylation, or crosslinking were effective methods for improving the hydration properties of JJRDF, and these methods can expand its applications in protein gel-based food. Moreover, the improvement in textural quality via the addition of JJRDFs expands the applications of HA-EPGs in food, medicine, biotissue engineering, skin-care masks, etc. However, the mechanisms underlying the structural changes in HA-EPGs, mechanical rheology, and syneresis test should be carried out in future research. The effects of these modifications on the functionalities, safety, and feasibility of JJRDFs need to be investigated, too.

## 4. Materials and Methods

### 4.1. Materials

Jujube (*Ziziphus jujuba* Mill.) and egg white protein powder expeller were purchased from Maishu Red Jujube Processing Factory, Linxian, China, and Hongxing Egg Product Factory, Hebi city, China, respectively. The egg white powder contained a protein content of >98.5 g/100 g. Cellulase (from Aspergillus niger, 1.0 × 10^6^ U/g), xylanase (from Trichoderma Vride G, 5 × 10^3^ U/g), α-amylase (from Bacillus licheniformis, 5 × 10^4^ U/g), pepsin (from porcine stomach mucosa), and trypsin (from bovine pancreas, 1:250 U/mg) were purchased from Nanning Bohui Biological Reagent Factory, Nanning, China. Propylene oxide, thiosalicylic acid, sodium chloride, etc., were analytically pure and purchased from Jinjiang Chemical and Reagent Factory, Tianjin, China.

### 4.2. Extraction of JJRDF

The raw jujube juicing residue was ultrafinely ground to pass through a 120-mesh sieve (MZ-B3, Hanyang Sifter Instrument Co., Wuhan, China) [24]. The sieved jujube juicing residue (20 g) was placed into a triangular flask (1 L), and 500 mL of the phosphate buffer (0.1 mol/L, pH 5.5) and 60 mg of α-amylase were poured into the flask. Then, the mixture was thoroughly shaken at 275 rpm and 90 °C using a GWXJ-B thermostatic water bath oscillator (Hanyang Food Processing Instrument Factory, Wuhan, China). After 150 min, 0.1 mol/L of NaOH was used to increase the pH value of the mixture to 9.0 ± 0.1, and Alcalase (40 mg) was added. The mixture in the triangular flask was shaken (275 rpm and 50 °C) for 2 h. Then, 0.1 mol/L of HCl was used to adjust the pH value of the mixture to 2.0 ± 0.1, and amyloglucosidase (60 mg) was added. After 2.5 h of shaking (275 rpm and 60 °C), the mixture in the triangular flask was placed in a 100 °C water bath for 15 min, and then cooled, with further filtering carried out with ARWtman1107-C04 paper. The residue on the paper was washed using 95% ethanol (*v*/*v*, 100 mL) and dried for 12 h using a FGZG-I-6 blast dryer (Deyang Food Drying Instrument Factory, Deyang, China) to obtain jujube juicing residue dietary fiber (JJRDF).

### 4.3. Xylanase and Cellulase Hydrolysis

JJRDF (15 g), 300 mL of deionized water, 80 mg of xylanase, and cellulase (60 mg) were poured into a triangular flask (1 L) and thoroughly mixed using an MIX-M vortex oscillator (Shanghai Pawen Instrument Co., Ltd., Shanghai, China) at 1200 rpm for 12 s [26]. HCl (0.1 mol/L) was used to adjust the pH value of the dispersions to 5.0 ± 0.1. After 150 min of stirring (275 rpm and 55 °C), the dispersion in the triangular flask was placed in a 100 °C water bath for 10 min and then cooled, with further filtering carried out using ARWTman1107-C04 paper. After hydration using the FGZG-I-6 blast dryer (50 °C, 6 h), xylanase and cellulase hydrolyzed jujube juicing residue dietary fiber (JJRDF-CXH) was obtained.

### 4.4. Carboxymethylation of JJRDF-CXH

Carboxymethylation of JJRDF-CXH was performed using the protocols of Zheng et al. [28]. The xylanase and cellulase hydrolysis and carboxymethylated jujube juicing residue dietary fiber (JJRDF-CXHC) was obtained.

### 4.5. Phosphate Crosslinking of JJRDF-CXH

JJRDF-CXH (8 g), 200 mL of deionized water, 20 mL of Na_5_P_3_O (1.6 mg/mL), and NaO_9_P_3_ (24 mg/mL) were poured into a triangular flask (0.5 L) and thoroughly mixed using the MIX-M vortex oscillator at 1200 rpm for 6 s [35]. The triangular flask was placed in the GWXJ-B thermostatic water bath oscillator, and NaOH (0.25 mol/L) was used to adjust the pH value of the dispersions to 11.0 ± 0.1. The reaction was then started. After 3 h of stirring (275 rpm, 45 °C), HCl (1 mol/L) was used to stop the reaction via adjusting the pH value to 7.0. ARWTman1107-C04 paper was used for the filtration of the reaction mixture. After 3 rounds of distilled water rinsing, the residue was dried (47 °C, 5 h) and collected to obtain xylanase and cellulase hydrolysis and phosphate crosslinking-treated jujube juicing residue dietary fiber (JJRDF-CXHPC).

### 4.6. Acetylation of JJRDF-CXH

The acetylation of JJRDF-CXH was performed following the same procedures reported by Xu et al. [31], and xylanase and cellulase hydrolysis and acetylated jujube juicing residue dietary fiber (JJRDF-CXHA) was obtained.

### 4.7. Preparation of Heat- and Alkaline-Induced Egg Protein Gels Fortified with JJRDFs

Egg white proteins (90 g) and 180 mL of deionized water were poured into a triangular flask (0.5 L) and adjusted to pH 8.2 with 0.1 mol/L of NaOH. The mixture was thoroughly mixed using the MIX-M vortex oscillator at 12,00 rpm for 5 s, and left at 4 °C for 8 h [39]. The egg white protein solution was divided into 6 beakers. Each beaker contained 30 mL of the egg white protein solution and 1–5 g/100 g of JJRDF, JJRDF-CXH, JJRDF-CXHPC, and JJRDF-CXHA was separately added and mixed thoroughly. The beakers were shaken at 90 ± 1.0 °C and 140 rpm for 3 min, then allowed to stand for 27 min. After cooling to room temperature, the beakers were placed at 4 °C. Eight hours later, egg protein gels formed via heat and alkaline (HA-EPG) fortified with JJRDF (HA-EPG/JJRDF), JJRDF-CXH (HA-EPG/ JJRDF-CXH), JJRDF-CXHPC (HA-EPG/JJRDF-CXHPC), and JJRDF-CXHA (HA-EPG/JJRDF-CXHA) were obtained, respectively.

### 4.8. Determination of Constituent

Determination of the chemical constituents of JJRDFs, including protein, moisture, fat, and ash content, was performed following the procedures of AOAC.920.39, AOAC.924.05, AOAC.92.05, and AOAC.955.04, respectively [39]. Insoluble fiber content, including cellulose, hemicellulose, and lignin content, was calculated based on acid and neutral detergent fibers, and insoluble acid lignin content, which were measured using the method of Chu et al. [40]. The determination of soluble fiber content was conducted using the AOAC.991.43 method [39], and the total fiber content was the sum of the soluble and insoluble fiber content.

### 4.9. Colour Difference and Surface Area Measurement

The color difference (Δ*E*) and surface area of samples were determined using a CD-HNI30 Precision Colorimeter (Color Spectrum Technology Co., Ltd., Hangzhou, China) and a Bettersize2600 Laser Particle Size Analyzer (Dandong Baxter Instrument Co., Ltd., Dandong, China), respectively. The Δ*E* between the modified JJRDFs and untreated JFDF was calculated by comparing their lightness (*L*), yellowness (*b*), and redness (*a*) [35].

### 4.10. Surface Microstructure Scanning

The surface microstructure scanning of dry JFDFs coated with gold (10 nm) was carried out on a JOLE-JSM-5070E electron microscope (Japan Electronics Co., Ltd., Beijing, China). Scanning was performed at an accelerating voltage of 10,000 V, and the picture was captured at 2000 × (measuring scale of 5 μm). Tests were conducted in triplicate, and the scanning electron micrographs were representative of the samples.

### 4.11. Fourier-Transformed Infrared Spectroscopy

Dry KBr (20 mg) was thoroughly mixed with the JJRDFs (1 mg) under an NL-3C Infrared baking lamp, then pressed into 1–2 mm sheets [31]. Those sheets were analyzed with a Fourier-transform infrared (FT-IR) spectrometer (LIDA-20, Hengchuanglida Precision Instrument Co., Ltd., Tianjin, China) at a wavenumber range of 4000–400 cm^–1^, respectively. Tests were conducted in triplicate, and the average value of the transmittance was obtained.

### 4.12. Physicochemical Properties

#### 4.12.1. Ability to Retain Water

In a centrifuge tube, approximately 1 g of JJRDFs (*M*_0_) was mixed with 30 mL of deionized water (dH_2_O) and stirred at 115 rpm and 25 ± 1 °C using an ET-0016B thermostatic vibrator (Nantong Vibration Shaker Co., Nantong, China) for 120 min [26]. After centrifugation at 3300× *g* using a TGL-16.5M centrifuge (Luxiangyi Laboratory Instrument Co., Ltd., Shanghai, China) for 15 min, the supernatant was discarded, and the residue in the tube was weighed (*M*_2_). The ability to retain water (ARW) was calculated using Equation (1):

(1) $$ARW\left(g/g\right)=\left(M_{1}-M_{0}\right)/M_{0}$$

#### 4.12.2. Expansion Volume in Water

Dry JJRDFs (approximately 2 g) were placed in a glass measuring cylinder and the volume was measured (*V*_0_). After adding 70 mL of dH_2_O to the measuring cylinder, it was left to stand at 25 ± 1 °C for 20 h. Then, the volume of the wet JJRDFs was measured (*V*_1_). The expansion volume in water (EVW) was calculated using Equation (2):

(2) $$EVW\left(g/g\right)=\left(V_{1}-V_{0}\right)/M_{0}$$

#### 4.12.3. Viscosity

Citing the procedures of Xu et al. [31], JJRDF dispersions (1 g/25 mL) were measured using a NV-2T digital display rotary viscometer (AMETEK–Brookfield Co., Ltd., Guangzhou, China) at 25 ± 1 °C with a viscosity range of 1–320 cP.

### 4.13. Gel Characteristics

#### 4.13.1. Optical Transparency and Water-Retention Ability of HA-EPGs

Following Lv et al. [37], the optical transparency of HA-EPGs in a glass cuvette was determined using a UV-1200 UV–Vis spectrophotometer (Mepida Instrument Co., Ltd., Shanghai, China) at 600 nm. Moreover, as per the modified procedures of Khemakhem et al. [36], a small piece of HA-EPG was weighed (*M*_1_) and placed in a glass centrifuge tube. The weight of the tube and gel sample was weighed (*M*_2_), and it was then centrifuged at 5100× *g* and 4 °C using a TGL-16.5M centrifuge (Luxiangyi Laboratory Instrument Co., Ltd., Shanghai, China) for 25 min. Next, the water above the gel was carefully removed using filter paper, and then the weight of the gel and tube was measured (*M*_3_). The ability of the gel sample to retain water was calculated using Equation (3):

(3) $$ARW\left(g/g\right)={(M}_{2}-M_{3})/M_{1}$$

#### 4.13.2. Water-Losing Rate During Freeze–Thaw of HA-EPGs

Citing the procedures from Lv et al. [37], the HA-EPGs (approximately 4 g) were weighted (*M*_1_) and sealed in a glass tube (50 mL). The gel in the tube was frozen at −16 °C for 18 h and then thawed at 25 °C for 10 h. The sample in the tube was subjected to this freeze−thaw cycle five times, and centrifugation was carried out at 3750× *g* using a TGL-16.5M centrifuge (Luxiangyi Laboratory Instrument Co., Ltd., Shanghai, China) for 10 min. The supernatant water was discarded and the residue was weighed (*M*_2_). The water-losing rate during each freeze−thaw cycle was calculated as follows:

(4) $$Water\text{-}losingrate\left(\%\right)=(M_{1}-M_{2})/M_{1}\times 100\%\;$$

#### 4.13.3. Textural Quality

As per Hou et al. [33], a TA.HD plusC Physical Properties Test Analyzer (STABLE MICRO SYSTEMS, London, UK) was used to measure the textural quality of the HA-EPGs. Chewability, hardness, adhesiveness, and cohesiveness were determined using a P/36 R probe operating in the TPA mode, followed by a post-test speed of 1 mm/s, compression rate of 55.2%, test speed of 1 mm/s, trigger force of 5 g, and pre-test speed of 2 mm/s.

### 4.14. Simulated Gastrointestinal Digestion

As described by Lee et al. [38], the simulated gastric digestive fluid consisted of sodium chloride (0.18 mol/L), ultrapure water (180 mL), and 0.40 mg/mL of pepsin. The simulated intestinal digestive juice was composed of NaHCO_3_ (0.625 g/mL), bile salt (3 g/100 mL), pancreatin (0.35 mg/mL), and 180 mL of ultrapure water. Then, 3 g of HA-EPGs was cut into small pieces measuring approximately 50 mm in diameter, and they were placed in a glass triangle flask. Next, 60 mL of ultrapure water and 150 mL of the simulative gastric fluid were added and mixed thoroughly. The flask was placed in an ET-0016B thermostatic vibrator at 37 °C, with a shaking rate of 140 rpm. After 60 min, 180 mL of the simulated intestinal hydrolysis fluid was added and stirred at 37 °C for 2 h. To stop hydrolysis, the digestion solution in the flask was boiled for 6 min and then cooled to room temperature using running water. During the gastrointestinal hydrolysis, 0.75 mL reaction fluid was pipetted and centrifuged at 7150× *g* using the H1750R centrifuge (Xiangyi Centrifuge Development Co., Ltd., Changsha, China) for 15 min. The free amino acid concentration in the supernatant was quantified according to the *o*-phthalaldehyde (OPA) method [41].

### 4.15. Statistical Analysis

Tests were conducted in triplicate, and the statistical analysis (mean ± SD) was conducted using V.17.4 SPSS software (International Business Machines Corporation, Chicago, IL, USA). Statistically differences were analyzed via analysis of variance (ANOVA), and the Shapiro–Wilk test was used for normality tests. Significant difference was analyzed using Duncan’s multiple comparisons, and the threshold for significant differences (*p* < 0.05) was 95%.

## Figures and Tables

**Table 1:** Influences of different dual modifications on the chemical constitute, color, surface area, and hydration properties of jujube juicing residue dietary fiber.

Proximate Composition	JJRDF	JJRDF-CXHA	JJRDF-CXHC	JJRDF-CXHPC
Moisture (g/100 g)	7.47 ± 0.20 c	5.99 ± 0.24 c	5.95 ± 0.37 c	6.46 ± 0.17 c
Fat (g/100 g)	1.28 ± 0.09 c	1.12 ± 0.09 c	1.22 ± 0.08 c	1.41 ± 0.03 c
Protein (g/100 g)	2.19 ± 0.21 c	2.07 ± 0.09 c	1.89 ± 0.08 c	1.79 ± 0.07 c
Ash (g/ 100 g)	1.38 ± 0.08 d	1.74 ± 0.08 d	4.22 ± 0.27 c	2.89 ± 0.09 c
TDF (g/ 100 g)	75.38 ± 2.28 d	76.93 ± 4.82 c	77.43 ± 4.24 c	78.09 ± 3.58 c
IDF (g/100 g)	69.72 ± 4.52 c	65.92 ± 1.79 d	60.47 ± 2.61 e	60.56 ± 4.95 e
SDF (g/100 g)	5.66 ± 0.11 e	11.01 ± 2.56 d	16.96 ± 0.42 c	17.53 ± 2.32 c
Hemicellulose (g/100 g)	43.56 ± 3.47 c	30.28 ± 3.17 d	29.38 ± 2.77 d	30.99 ± 3.67 d
Cellulose (g/100 g)	16.53 ± 0.55 c	9.85 ± 0.08 d	10.79 ± 0.34 d	10.73 ± 1.05 d
Lignin (g/100 g)	9.63 ± 0.39 c	6.89 ± 3.11 d	8.79 ± 2.66 c	5.97 ± 0.39 e
D_3,2_ (μm)	116.47 ± 4.71 c	76.71 ± 2.07 e	59.38 ± 3.35 f	95.23 ± 4.05 d
Specific surface area (m^2^∙kg^−1^)	64.53 ± 3.74 f	105.15 ± 4.74 d	167.35 ± 4.42 c	86.44 ± 2.95 e
*L*	53.8 ± 1.02 c	40.75 ± 3.13 d	36.95 ± 2.44 d	38.56 ± 3.34 d
*a*	7.95 ± 0.23 d	10.57 ± 0.32 c	11.75 ± 0.37 c	9.36 ± 0.27 c
*b*	11.32 ± 0.26 e	14.26 ± 0.26 d	19.54 ± 0.37 c	15.08 ± 1.42 d
Δ*E*	Control	13.63	19.13	15.76
Ability to retain water (g/g)	6.02 ± 0.34 d	7.17 ± 0.36 d	12.83 ± 0.56 c	14.75 ± 0.23 c
Expansion volume in water (mL/g)	5.60 ± 0.20 e	8.42 ± 0.40 d	10.40 ± 0.20 c	9.80 ± 0.20 c
Viscosity (cP)	7.01 ± 0.33 f	10.56 ± 0.08 e	18.46 ± 0.36 c	13.92 ± 0.43 d

JJRDF, jujube juicing residue dietary fiber; JJRDF-CXHA, JJRDF hydrolyzed with cellulase and xylanase hydrolysis and acetylation; JJRDF-CXHC, JJRDF modified via cellulase and xylanase hydrolysis and carboxymethylation; JJRDF-CXHPC, JJRDF modified via cellulase and xylanase hydrolysis combined with phosphate crosslinking; TDF, total dietary fiber; SDF, soluble dietary fiber; IDF, insoluble dietary fiber. D_3,2_, the Sauter mean diameter of JJRDFs. Color indexes including *L*, *a*, and *b* are representative of rightness, redness, and yellowness of fibers, respectively, and Δ*E* means the difference in color between JJRDF and the modified JJRDFs. Different lowercase letters (c–f) in the same row indicate significant differences (*p* < 0.05).

**Table 2:** Effects of addition of JJRDFs (3 g/100 g) on the color of heat- and alkaline-induced egg protein gel.

Gels	*L*	*a*	*b*	Δ*E*
HA-EPG	89.37 ± 1.19 a	–1.13 ± 0.07 c	13.34 ± 1.02 c	Control
HA-EPG/JJRDF	77.98 ± 6.67 c	3.36 ± 0.07 b	15.16 ± 0.54 b	12.378
HA-EPG/JJRDF-XCHC	78.90 ± 3.33 c	4.13 ± 0.12 ab	15.30 ± 1.07 b	11.88
HA-EPG/JJRDF-XCHPC	78.18 ± 2.67 c	5.46 ± 0.19 a	22.30 ± 0.33 a	15.78
HA-EPG/JJRDF-XCHA	83.08 ± 1.33 b	3.35 ± 0.33 b	15.96 ± 0.45 b	8.16

Different small letters (a–c) in the same column indicate significant differences (*p* < 0.05).

**Table 3:** Effects of different dose of JJRDFs on the textural properties of heat- and alkaline-induced egg protein gel.

Gels	Amount (g/100 g)	Hardness (g)	Adhesiveness	Springiness	Cohesiveness	Gumminess	Chewiness (g)
HA-EPG	0	93.16 ± 4.85 f	−47.32 ± 1.38 d	0.91 ± 0.02 a	0.77 ± 0.02 a	83.99 ± 5.72 e	80.14 ± 3.77 e
HA-EPG/JJRDF	1	95.87 ± 3.40 f	−34.92 ± 0.78 b	0.91 ± 0.01 a	0.81 ± 0.01 a	69.07 ± 3.65 f	78.65 ± 4.53 e
	3	109.87 ± 7.56 e	−33.41 ± 0.18 b	0.90 ± 0.00 a	0.77 ± 0.01 a	93.58 ± 7.11 d	97.38 ± 0.77 d
	5	127.93 ± 7.47 d	−26.21 ± 0.35 a	0.89 ± 0.00 a	0.82 ± 0.02 a	99.33 ± 0.67 d	101.01 ± 4.75 d
HA-EPG/JJRDF-XCHC	1	116.53 ± 4.99 e	−40.26 ± 1.94 c	0.87 ± 0.01 a	0.79 ± 0.02 a	98.25 ± 6.75 d	94.68 ± 7.34 d
	3	141.82 ± 9.11 c	−36.53 ± 2.66 b	0.89 ± 0.03 a	0.81 ± 0.02 a	124.56 ± 7.99 d	111.39 ± 6.42 cd
	5	177.93 ± 5.26 b	−39.84 ± 4.73 c	0.90 ± 0.01 a	0.79 ± 0.01 a	159.17 ± 9.84 b	118.01 ± 3.99 c
HA-EPG/JJRDF-XCHPC	1	79.44 ± 4.59 g	−48.78 ±3.29 d	0.91 ± 0.02 a	0.74 ± 0.02 a	94.79 ± 6.94 d	72.56 ± 8.33 e
	3	148.83 ± 11.55 c	−43.95 ±7.54 d	0.90 ± 0.01 a	0.81 ± 0.01 a	147.66 ± 1.48 c	149.62 ± 9.12 b
	5	201.41 ± 8.83 a	−46.00 ±3.42 d	0.88 ± 0.02 a	0.82 ± 0.01 a	173.56 ± 7.48 a	177.99 ± 7.58 a
HA-EPG/JJRDF-XCHA	1	117.28 ± 11.06 e	−36.39 ± 1.58 b	0.87 ± 0.02 a	0.81 ± 0.02 a	90.01 ± 4.55 d	87.96 ± 4.98 d
	3	142.12 ± 7.43 c	−30.23 ± 0.72 b	0.89 ± 0.01 a	0.83 ± 0.01 a	120.75 ± 9.58 d	105.44 ± 9.72 d
	5	157.67 ± 5.79 c	−39.21 ± 1.15 b	0.93 ± 0.01 a	0.81 ± 0.02 a	139.88 ± 9.77 c	123.55 ± 9.13 c

Different small letters (a–g) in the same column indicate significant differences (*p* < 0.05).

## Data Availability

The original contributions of this study are included in the article; further inquiries can be directed to the corresponding author.

## Abbreviations

The following abbreviations are used in this manuscript:

JJRDF	Jujube juicing residue dietary fiber
JJRDF-CXHC	Jujube juicing residue dietary fiber modified by cellulase and xylanase hydrolysis coupled with carboxymethylation
JJRDF-CXHA	Jujube juicing residue dietary fiber modified by cellulase and xylanase hydrolysis coupled with acetylation
JJRDF-CXHPC	Jujube juicing residue dietary fiber modified by cellulase and xylanase hydrolysis coupled with phosphate crosslinking
HA-EPG	Heat- and alkaline-induced egg protein gel
ARW	Ability to retention water
